# Tamizaje neonatal en Colombia: la experiencia de un programa privado en Bogotá

**DOI:** 10.7705/biomedica.6911

**Published:** 2024-03-31

**Authors:** Jaime E. Bernal, Martha Lucía Tamayo, Ignacio Briceño, Escilda Benavides

**Affiliations:** 1 Facultad de Medicina, Universidad del Sinú, sede Cartagena, Colombia Universidad del Sinú Universidad del Sinú Cartagena Colombia; 2 Pregen Colombia, Bogotá, D.C., Colombia Pregen Pregen Bogotá, D.C. Colombia; 3 Instituto de Genética Humana, Pontificia Universidad Javeriana, Bogotá, D.C., Colombia Pontificia Universidad Javeriana Pontificia Universidad Javeriana Bogotá, D.C. Colombia; 4 Facultad de Medicina, Universidad de la Sabana, Chía, Colombia Universidad de la Sabana Universidad de la Sabana Chía Colombia; 5 Departamento de Ciencias Básicas, Universidad del Sinú, sede Cartagena, Colombia Universidad del Sinú Universidad del Sinú Cartagena Colombia

**Keywords:** tamizaje neonatal, recién nacido, hemoglobinas, hipotiroidismo congénito, Colombia, Neonatal screening, infant, newborn, hemoglobins, congenital hypothyroidism, Colombia

## Abstract

**Introducción.:**

En Colombia, el primer programa de tamizaje neonatal, PREGEN, inició labores en el sector privado de Bogotá en 1988. En este artículo se presentan los resultados obtenidos en los últimos años, que, dada la carencia de estos estudios en el país, pueden servir para evaluar la frecuencia de aparición de los trastornos congénitos evaluados y estimar cuáles de ellos deben ser objeto de tamizaje neonatal a nivel nacional.

**Objetivos.:**

Reportar los resultados del programa de tamizaje PREGEN entre el 2006 y el 2019.

**Materiales y métodos.:**

Para este análisis se examinaron las bases de datos y otros documentos informativos de PREGEN para el periodo 2006-2019.

**Resultados.:**

Uno de cada 164 recién nacidos tamizados en el programa PREGEN en Bogotá presentó una variante anormal de la hemoglobina y uno de cada 194 es portador de hemoglobina S. Los siguientes dos trastornos más frecuentes encontrados fueron la deficiencia de la enzima glucosa-6-fosfato deshidrogenasa (frecuencia 1:2.231) y el hipotiroidismo congénito (frecuencia 1:3.915).

**Conclusiones.:**

Las hemoglobinopatías mostraron ser uno de los desórdenes monogénicos más comunes, seguidos por la deficiencia de glucosa-6-fosfato deshidrogenasa y el hipotiroidismo congénito. Se calcula que cerca de 400 millones de personas en el mundo están afectadas por la deficiencia de glucosa-6-fosfato deshidrogenasa, por lo cual es la enzimopatía más común en el mundo. Como ambos desórdenes son más frecuentes en poblaciones de origen africano y confieren algún grado de resistencia a la malaria, es de prever que su tamizaje debe ser de mayor importancia en las zonas con ancestros africanos en Colombia.

## Abstract (en)

**Introduction.:**

The first neonatal screening program in Colombia - PREGEN - was set up in the medical private sector of Bogotá in 1988. We report the results from recent years that, given the scarcity of similar information in our country, may help estimate the frequency of the evaluated neonatal disorders and which ones should be included in the neonatal screening programs in our country.

**Objective.:**

To describe the results of PREGEN's newborn screening program between 2006 and 2019.

**Materials and methods.:**

We analyzed databases and other informative documents preserved in PREGEN from the 2006-2019 period.

**Results.:**

One in every 164 newborns screened in our program had an abnormal hemoglobin variant, and one in every 194 carried some hemoglobin S variant. Glucose-6- phosphate dehydrogenase deficiency and congenital hypothyroidism are next as the more common disorders.

**Conclusions.:**

Abnormal hemoglobin causes the most frequent monogenic disorder in the world. Glucose-6-phosphate dehydrogenase deficiency is the most common enzymopathy affecting nearly 400 million individuals worldwide. Since both disorders are more common in people of African descent and confer some resistance to malaria, we believe that screening for both disorders may be more relevant in the areas with African ancestry in our country.

El término tamizaje neonatal se refiere a cualquier tipo de prueba que se aplique a recién nacidos con miras a detectar aquellos individuos que puedan desarrollar alguna enfermedad y con ello, practicar las pruebas diagnósticas que se requieran para confirmar o descartar el caso. Un programa de tamizaje neonatal incluye un laboratorio adecuado para adelantar las pruebas requeridas en muestras biológicas tomadas en las primeras horas o días de vida del recién nacido. Las muestras se procesan en el menor tiempo posible por personal calificado que, además de la toma de muestras, se encarga de los análisis de laboratorio, el seguimiento y el tratamiento oportuno de los pacientes.

El tamizaje neonatal ha sido útil para reducir la morbimortalidad neonatal e infantil. En menos de 50 años, la metodología del tamizaje ha evolucionado desde una pequeña muestra de sangre u orina con análisis bioquímicos relativamente sencillos, hasta un trabajo de laboratorio más sofisticado que incluye secuenciación genómica de próxima generación para la detección de múltiples condiciones. Por esta razón, se requieren consensos sobre los métodos de laboratorio por usar y las enfermedades que se recomiendan tamizar ^1^^,^^2^. En una anterior comunicación se reportaron los resultados del tamizaje auditivo neonatal mediante emisiones otacústicas ^3^.

En Colombia, el primer programa de tamizaje neonatal, denominado Prevención y Genética (PREGEN), inició labores en el sector privado en Bogotá el 19 de febrero de 1988. En este artículo se presentan los resultados obtenidos entre el 2006 y el 2019 que, dada la carencia de estos estudios en el país, pueden servir para calcular la frecuencia de aparición de trastornos genéticos congénitos y estimar así cuáles de ellos deben ser objeto de tamizaje neonatal en Colombia.

## Material y métodos

PREGEN es un programa privado y voluntario que se ofrece en diversas clínicas de Bogotá a los padres de recién nacidos. Durante el 2006 al 2019, PREGEN tomó muestras de recién nacidos de las clínicas Fundación Santafé, Country, Palermo, de la Mujer, Marly y La Colina y procesó varias de estas (con excepción de las de la clínica del Country) para evaluar la hormona tiroestimulante (TSH) neonatal, requerida por ley. PREGEN mantiene una base de datos, que se actualiza mensualmente, donde se registra el total de pacientes tamizados por prueba y aquellos que resultaron positivos, una vez confirmado el diagnóstico. Dado que no todas las pruebas se implementaron en el mismo momento (principalmente debido a la disponibilidad comercial de los reactivos), la cantidad de recién nacidos tamizados entre el 2006 y el 2019 es diferente para cada año (cuadro 1).

**Cuadro 1:** Número de recién nacidos tamizados

Enfermedad o técnica	Recién nacidos tamizados (n)
Hemoglobinopatías	40.182
Hipotiroidismo congénito	129.210
Deficiencia de biotinidasa	30.834
Galactosemia	30.608
Hiperplasia suprarrenal congénita	30.298
Fibrosis quística	10.074
Deficiencia de glucosa-6-fosfato deshidrogenasa	8.926
Fenilcetonuria	2.422
Espectrometría de masas en tándem	13.714
Total	296.268

Las hemoglobinopatías se evaluaron por cromatografía líquida de alta eficacia (HPLC) con el equipo GeneSys2™ (Trinity Biotech Assay). Las pruebas para detección de fibrosis quística, galactosemia, fenilcetonuria, hipotiroidismo congénito, hiperplasia suprarrenal congénita, deficiencia de biotinidasa y deficiencia de glucosa-6-fosfato deshidrogenasa (G6PD) se realizaron con el analizador de inmunoensayos enzimáticos Delfia™ (Perkin Elmer).

Los controles internos de las pruebas son muestras de valores conocidos provistas con los reactivos y se corren al tiempo con las muestras de los pacientes. Los controles externos son llevados a cabo por varias entidades (cuadro 2).

**Cuadro 2:** Entidades que realizan los controles de calidad externos.

Entidad	Prueba	Periodicidad	Muestras analizadas
Instituto Nacional de Salud	TSH	Cuatro por año	Cada envío, dos lotes y en cada lote seis muestras
Secretaría de Salud (supervisión indirecta)	TSH	Mensual	50 muestras mensuales
CDC de Atlanta-PT Control no valorado (mide la reproducibilidad de todas las pruebas)	TSH	Cuatro por año	Cinco muestras por prueba
	17OHP		
	GAL		
	BTD		
	G6PD		
	IRT		
	PKU, en espera,		
CDC de Atlanta, control de calidad (control valorado, mide exactitud)	TSH	Cuatro por año	15 muestras por prueba
	17OHP		
Programa de evaluación externa de calidad de la Fundación Bioquímica de Argentina	TSH	Cuatro por año	Cada envío, dos lotes

## Resultados

Entre los 40.182 recién nacidos tamizados para hemoglobinopatías se encontraron 245 con hemoglobinas anormales, 194 con hemoglobina S, seguidos por aquellos con hemoglobina C (cuadro 3). En otras palabras, se estima que uno de cada 164 recién nacidos tamizados en Bogotá por PREGEN presentaba una variante anormal de la hemoglobina y uno de cada 194 era portador de hemoglobina S.

**Cuadro 3:** Casos detectados de hemoglobinas anormales

Hemoglobinopatías	Casos detectados (N = 40.182)	Frecuencia (n)
HBS	194	1:207
HBC	34	1:1.181
HBE	2	1:20.091
HB Barts	3	1:13.394
Variante de cadena A	1	1:40.182
Variante de cadena B	10	1:4.018
Variante de cadena D	1	1:40.182
Total	245	1:164

TSH: hormona estimulante de la tiroides; 17OHP: 17-hidroxiprogesterona; GALT: galactosa-1-fosfato uridiltransferasa; G6PD: glucosa-6-fosfato deshidrogenasa; IRT: tripsinógeno inmunorreactivo humano para detectar fibrosis quística; PKU: prueba de Guthrie para detectar fenilcetonuria

En el cuadro 4 se encuentra el número de casos detectados y el número total de pacientes tamizados para cada una de las siete pruebas. Se observa que la deficiencia de G6PD y el hipotiroidismo congénito fueron los trastornos detectados con mayor frecuencia en este grupo, de 1:2.231 para G6PD y de 1:3.915 para hipotiroidismo. Los resultados de los casos analizados por espectrometría de masas en tándem se encuentran en el cuadro 5. Se detectaron ocho casos entre 13.714 recién nacidos tamizados, lo cual indica que por este método se detectan trastornos en cerca del 0,06 % de los individuos analizados. Los desórdenes más frecuentes fueron la enfermedad de orina con olor a jarabe de arce y la acidemia metilmalónica.

**Cuadro 4:** Casos detectados de otros trastornos

Analito	Casos detectados (n)	Frecuencia (n)
TSH	33 (129.210)	1:3.915
Biotinidasa	1 (30.834)	1:30.834
17OHP	1 (30.298)	1:30.298
GALT	1 (30.608)	1:30.608
G6PD	4 (8.926)	1: 2.231
IRT	0 (10.074)	------
PKU	0 (2.422)	------

TSH: hormona estimulante de la tiroides; 17OHP: 17-hidroxiprogesterona; GALT: galactosa-1-fosfato uridiltransferasa; G6PD: glucosa-6-fosfato deshidrogenasa; IRT: tripsinógeno inmunorreactivo humano para detectar fibrosis quística; PKU: fenilcetonuria

**Cuadro 5:** Casos detectados mediante espectrometría de masas en tándem

Espectrometría de masas en tándem	Casos detectados (N = 13.714)	Frecuencia (n)
Orina con olor a jarabe de arce	3	1:4.571
Acidemia metilmalónica	2	1:6.857
Fenilcetonuria	1	1:13.714
Deficiencia de carnitina	1	1:13.714
Hipermetioninemia	1	1:13.714

Finalmente, en el cuadro 6 se registra el número de repeticiones que, por diversos motivos, se hizo para las distintas pruebas de laboratorio. En general, la frecuencia de estas repeticiones es baja, igual o menor a 1 %. Se destacan los análisis de hemoglobinas y fenilcetonuria como los de más alta frecuencia.

**Cuadro 6:** Número de repeticiones requeridas para la confirmación de cada prueba

Entidad	Repeticiones / Total de tamizados (%)	
Hipotiroidismo congénito	253 / 129.210	(0,20)
Hemoglobinopatías	334 / 40.182	(0,83)
Deficiencia de biotinidasa	136 / 30.834	(0,44)
Hiperplasia suprarrenal congénita	48 / 30.298	(0,16)
Galactosemia	89 / 30.608	(0,29)
Deficiencia de glucosa-6-fosfato deshidrogenasa	51 / 8.926	(0,57)
Fibrosis quística	13 / 10.074	(0,13)
Fenilcetonuria	24 / 2.422	(1,00)

## Discusión

Lo primero que salta a la vista entre 296.268 tamizajes de este estudio es que los trastornos más frecuentemente encontrados fueron las hemoglobinas anormales (1:164), seguidos de la deficiencia de G6PD (1:2.231) y el hipotiroidismo congénito (1:3.915). Otras dos entidades tienen frecuencias menores a 1:10.000: la enfermedad de orina con olor a jarabe de arce (1:4.571) y la acidemia metilmalónica (1:6.857); otros trastornos tienen una frecuencia superior de 1:13.000 como la fenilcetonuria, la deficiencia de carnitina y la hipermetioninemia. Los restantes trastornos evaluados (deficiencia de biotinidasa, hiperplasia suprarrenal congénita y galactosemia) se presentan en alrededor de uno por cada 30.000 recién nacidos.

Las hemoglobinopatías son los desórdenes monogénicos más comunes en el mundo ^4^, lo cual se confirma en este estudio ya que fue el hallazgo más frecuente entre todos los estudiados. La deficiencia de G6PD es una predisposición hereditaria a la hemólisis, ligada al cromosoma X, debida a una mutación en el gen que codifica para la enzima. Se calcula que cerca de 400 millones de personas están afectadas, por lo cual es la enzimopatía más común en el mundo ^5^. Las hemoglobinopatías y la deficiencia de G6PD son más frecuentes en las poblaciones de origen africano y confieren algún grado de resistencia a la malaria ^6^. Bogotá no es zona de transmisión de malaria y apenas tenía 1,4 % (de 7,59 millones de habitantes en el 2019) que se definían como de origen africano ^7^. Por lo tanto, es de prever que el tamizaje para ambos desórdenes debe tener mayor impacto médico y social en tierras bajas y zonas costeras donde existe el vector de la malaria y se asienta la mayoría de los afrodescendientes colombianos.

En resumen, este estudio resalta la importancia de un programa completo de tamizaje neonatal y lo valioso que sería extenderlo a todo el territorio nacional, en especial en un país como Colombia donde, entre el 1998 y el 2019, se observó un crecimiento del 32,6 % en el número de nacimientos, pasando de 140.120 en 1998 a 185.792 en el 2019. El registro de Bogotá mostró que se presentaron 30.552 nacimientos por año.

Un buen programa de tamizaje se caracteriza no sólo por la calidad del laboratorio y la inclusión de enfermedades importantes para esa región, sino por el seguimiento que haga del recién nacido y su familia. Por ello, se debe mantener un excelente programa de toma de muestras, una rapidez adecuada en su procesamiento, resultados de calidad y, sobre todo, un personal médico calificado que se encargue del seguimiento y búsqueda del tratamiento oportuno de los bebes que presenten alguna alteración.

Es de resaltar que, en la cohorte analizada en Bogotá, el trastorno más frecuente fueron las hemoglobinopatías (1:164), seguidas de la deficiencia de G6PD (1:2.231) y, en tercer lugar, el hipotiroidismo congénito (1:3.915). Se detectaron otros trastornos menos frecuentes, pero que requieren igual atención. Deben tenerse en cuenta los criterios clásicos para la inclusión de enfermedades en programas masivos de tamizaje neonatal -que contemplan elementos éticos, sociales y financieros ^8^-, así como la frecuencia de repeticiones, que incide en el costo total de las pruebas, y la creciente disponibilidad de técnicas de laboratorio en el mercado ^9^. Para efectos comparativos se incluyen referencias de dos estudios previos en Colombia ^10^^,^^11^.
